# Real World Implementation of a Transdiagnostic Risk Calculator for the Automatic Detection of Individuals at Risk of Psychosis in Clinical Routine: Study Protocol

**DOI:** 10.3389/fpsyt.2019.00109

**Published:** 2019-03-13

**Authors:** Paolo Fusar-Poli, Dominic Oliver, Giulia Spada, Rashmi Patel, Robert Stewart, Richard Dobson, Philip McGuire

**Affiliations:** ^1^Early Psychosis: Interventions and Clinical-detection (EPIC) Lab, Department of Psychosis Studies, Institute of Psychiatry, Psychology & Neuroscience, King's College London, London, United Kingdom; ^2^OASIS Service, South London and Maudsley National Health Service (NHS) Foundation Trust, London, United Kingdom; ^3^Department of Brain and Behavioral Sciences, University of Pavia, Pavia, Italy; ^4^National Institute for Health Research, Maudsley Biomedical Research Centre, South London and Maudsley National Health Service (NHS) Foundation Trust, London, United Kingdom; ^5^Department of Psychosis Studies, Institute of Psychiatry, Psychology & Neuroscience, King's College London, London, United Kingdom; ^6^Institute of Psychiatry, Psychology and Neuroscience, King's College London, London, United Kingdom; ^7^Institute of Health Informatics Research, University College London, London, United Kingdom; ^8^Health Data Research UK London, University College London, London, United Kingdom

**Keywords:** psychosis, schizophrenia, risk, transdiagnostic, prevention

## Abstract

**Background:** Primary indicated prevention in individuals at-risk for psychosis has the potential to improve the outcomes of this disorder. The ability to detect the majority of at-risk individuals is the main barrier toward extending benefits for the lives of many adolescents and young adults. Current detection strategies are highly inefficient. Only 5% (standalone specialized early detection services) to 12% (youth mental health services) of individuals who will develop a first psychotic disorder can be detected at the time of their at-risk stage. To overcome these challenges a pragmatic, clinically-based, individualized, transdiagnostic risk calculator has been developed to detect individuals at-risk of psychosis in secondary mental health care at scale. This calculator has been externally validated and has demonstrated good prognostic performance. However, it is not known whether it can be used in the real world clinical routine. For example, clinicians may not be willing to adhere to the recommendations made by the transdiagnostic risk calculator. Implementation studies are needed to address pragmatic challenges relating to the real world use of the transdiagnostic risk calculator. The aim of the current study is to provide *in-vitro* and *in-vivo* feasibility data to support the implementation of the transdiagnostic risk calculator in clinical routine.

**Method:** This is a study which comprises of two subsequent phases: an *in-vitro* phase of 1 month and an *in-vivo* phase of 11 months. The *in-vitro* phase aims at developing and integrating the transdiagnostic risk calculator in the local electronic health register (primary outcome). The *in-vivo* phase aims at addressing the clinicians' adherence to the recommendations made by the transdiagnostic risk calculator (primary outcome) and other secondary feasibility parameters that are necessary to estimate the resources needed for its implementation.

**Discussion:** This is the first implementation study for risk prediction models in individuals at-risk for psychosis. Ultimately, successful implementation is the true measure of a prediction model's utility. Therefore, the overall translational deliverable of the current study would be to extend the benefits of primary indicated prevention and improve outcomes of first episode psychosis. This may produce significant social benefits for many adolescents and young adults and their families.

## Introduction

**Figure 1:**
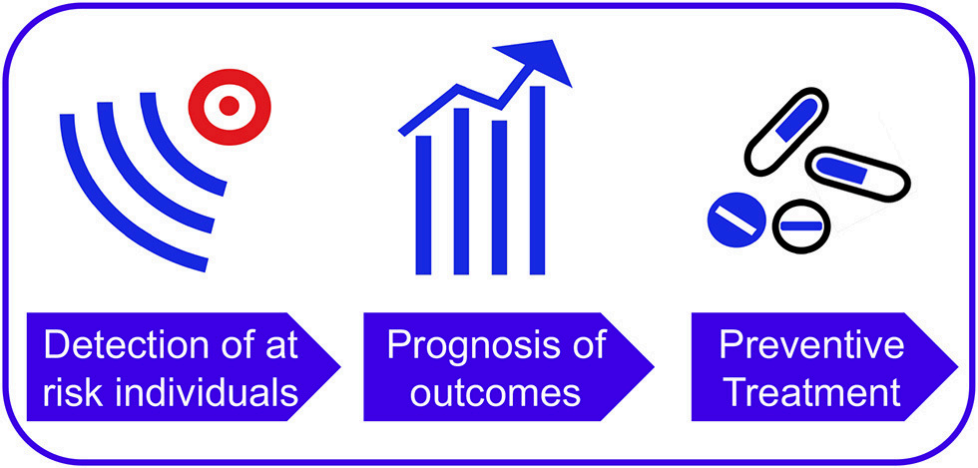
Core clinical and research components for effective prevention of psychosis.

Outcomes of psychotic disorders are associated with high personal, familial, societal, and clinical burden (1). There is thus an urgent clinical and societal need for improving outcomes of psychosis (1). The past two decades of clinical research have opened new opportunities for ameliorating outcomes of psychosis by intervening during its early clinical stages (1), in individuals at Clinical High Risk for psychosis [CHR-P (2)] -such as those meeting the At Risk Mental State criteria (3) or other similar criteria (4)-. This type of intervention is termed as “primary indicated prevention.” CHR-P individuals display subtle symptoms and overall functional impairment (5) that are due to the accumulation of several risk factors for psychosis (6, 7). In the wake of these issues (8), they seek help at specialized CHR-P clinics (9), where they receive a comprehensive psychometric assessment in the context of a clinical interview (10). Overall, the prognostic performance of this assessment is considered to be good [except for their use as screening tools in the general population (11, 12)] and comparable to that of similar prognostic measurements that are employed in organic medicine (13). Under those circumstances, CHR-P individuals have a 20% [see eTable 4 in (14)] probability of developing emerging psychotic disorders [but not other non-psychotic disorders (15, 16)] over a relatively short period of 2 years. Primary indicated prevention in CHR-P individuals has the unique potential to alter the course of psychosis and reduce the duration of untreated psychosis, although there is some uncertainty with respect to the true effectiveness of available treatments (17–21). An additional potential advantage is that secondary prevention in CHR-P who will develop the disorder can reduce the duration of untreated psychosis and ameliorate the severity of the disorder (1, 22). As summarized in Figure 1, the potential real world impact of the CHR-P paradigm for improving the outcomes of psychotic disorders is determined by the successful and stepped integration of the following key components:

- Efficient detection of individuals at-risk for psychosis;
- Accurate prognosis of outcomes;
- Effective preventive treatment.

illustrated in Figure 1, the first rate-limiting step for improving outcomes of psychosis through the CHR-P paradigm is the detection of individuals who are at risk for psychosis. In fact, even the most accurate prognostic tool and the most effective preventive treatment would have little impact on improving the outcomes of psychosis without proper scalability to the vast majority of the at-risk population. Our lab (the Early Psychosis: Intervention and Clinical-detection, EPIC) has investigated the effectiveness of current detection strategies for identifying CHR-P individuals for the first time. These strategies are largely based on referrals to specialized CHR-P clinics (9) that are made on suspicion of psychosis-risk (23). Our local National Health Service (NHS) Trust, The South London And the Maudsley (SLaM), in partnership with King's College London and King's Health Partners, hosts one of the largest clinical services for CHR-P individuals worldwide: the Outreach And Support In South London (OASIS) (24). Established in 2001, the OASIS has emerged as a reference point for psychosis prevention in the UK and worldwide (24). The OASIS is detecting CHR-P individuals from the community, primary care, and secondary care through an extensive and ongoing outreach campaign, which has been fully established over the past years. Despite this outreach, we have found that OASIS' detection strategies are highly inefficient because only 5% of individuals diagnosed with a first episode of non-organic ICD-10 psychosis in SLaM had been detected at their CHR-P stage. This finding has clear-cut clinical implications. For example, NHS England, in April 2016 has implemented a new Access and Waiting Times-Standard for Early Intervention in psychosis, which requires that CHR-P are detected nationwide and treated rapidly (25, 26). Although it is now an NHS requirement that all suspected CHR-P patients who present to NHS Trusts are assessed and interviewed for a psychosis-risk state (13), such an approach is likely to miss the vast majority of those at risk. No alternatives are on the horizon. Intensifying the inefficient outreach campaigns currently adopted by CHR-P clinics is not viable because these campaigns are idiosyncratic and unstandardized (23, 27, 28), leading to a diluted transition risk and unreliable prognosis (11, 12). Front-line youth mental health services such as the Headspace initiative -as opposed to specialized CHR-P clinics such as the OASIS- are also expected to detect more at-risk individuals. Unfortunately, even youth mental health services can detect only 12% of first episode cases at the time of their CHR-P stage (29). It is thus clear that to extend the benefits of the CHR-P paradigm some innovative approaches are urgently needed (30).

To overcome these issues, we have developed a pragmatic, clinically-based, individualized, transdiagnostic risk calculator for the detection of individuals at risk of psychosis in secondary mental health care at scale (24). In a subsequent step, the calculator has been externally validated, demonstrating good prognostic accuracy (24). Yet, a good model's (external) performance is necessary but not sufficient to ensure a clinical use of a risk calculator. Implementation studies are first needed to address pragmatic challenges relating to the use of a risk calculator in clinical routine (31). These challenges may suggest adaptations to the original models to allow its usability in the real world. Ultimately, successful implementation is the true measure of a prediction model's utility (32). For example, the transdiagnostic risk calculator was developed on retrospective cohort data (24). As such, it is not known whether it can be used prospectively in the real world of NHS Trusts. Data that are necessary to run the calculator (age, gender, age by gender, ethnicity, and ICD-10 index diagnosis) may not be available or not accessible. Furthermore, clinicians' adherence to the recommendations made by the transdiagnostic risk calculator is unknown. This represents the critical barrier toward its scalability in clinical routine. We describe here the protocol for the implementation study of this transdiagnostic risk calculator in the NHS. To our best knowledge, this will be the first implementation study of a risk calculator for clinical routine in the CHR-P field.

The overall translational deliverable of the current study would be to extend the benefits of primary indicated prevention and improve outcomes of first episode psychosis. This, in turn, may produce significant social benefits and cost-saving to many adolescents and young adults, their families and the NHS.

## Methods

This is a feasibility study which will evaluate essential real world parameters associated with the implementation of an electronic, clinically-based, individualized, transdiagnostic risk calculator for the detection of individuals at risk and the prediction of psychosis in secondary mental health care. Obtaining these figures is a necessary step in order to accurately estimate the resources (e.g., staffing) needed for the routine clinical use of the calculator. There are two phases in this study: an initial *in-vitro* (1 month) testing which does not involve patients contact and a second *in-vivo* piloting (11 months, total study duration 12 months), which involves recruitment of SLaM patients. Before we present the study design, we will briefly summarize the core characteristics of the transdiagnostic risk calculator.

### Clinically-Based, Individualized, Transdiagnostic Risk Calculator for the Automatic Detection of Individuals at Risk of Psychosis in Secondary Mental Health Care

In a previous meta-analysis, we showed that secondary mental health care is the most frequent source of referrals to CHR-P services such as the OASIS (23). We additionally confirmed that the recruitment of individuals for CHR-P assessment through secondary mental health services is associated with the highest probability of developing psychosis (27). In fact, these individuals are more likely to have accumulated several risk factors for psychosis such as affective comorbidities, substance abuse and social deprivation (6). These findings are concurrent to the European Psychiatric Association guidelines, which recommend that CHR-P assessment should only be offered to individuals who are “already distressed by mental problems and seeking help for them” (33). The transdiagnostic risk calculator presented here is therefore in line with the clinical guidelines in the field.

**Figure 2:**
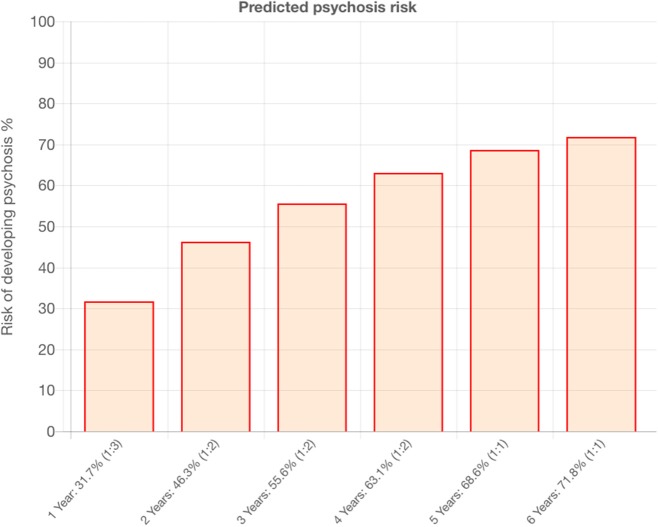
Example of outcomes produced by the transdiagnostic risk calculator that is under implementation in the current study.

Our calculator was developed and externally validated in a large clinical dataset of non-psychotic patients affected with non-organic mental disorders (*n* = 91,199), in the National Institute of Health Research (NIHR) Maudsley Biomedical Research Centre (BRC) Case Register. This register is electronic, because SLaM is paper-free, and all clinicians record their activity electronically on the Patient Journey System (PJS), as part of their clinical routine. PJS is a comprehensive record of all clinical information recorded throughout patients' journeys through SLaM NHS Trust services, including demographic and contact information, dates and other details of referrals and transfers, detailed clinical assessments, care plans and medication, clinical activity, and reviews. Anonymised information from PJS is subsequently used to create the Clinical Record Interactive Search (CRIS). CRIS allows researchers to search from PJS records. The details of the CRIS and the local electronic health record have been published previously (34–36). Therefore, this calculator leverages the potentials of e-Health innovations. The original study followed state-of-the-art guidelines for model development and validation (37). Thus, the external validation was done through a geographical split of the initial database in a derivation (Lambeth and Southwark SLaM boroughs, *n* = 33,820) and validation (Lewisham and Croydon SLaM boroughs, *n* = 54,716). The calculator showed good prognostic accuracy in the external validation, in terms of overall performance (*R*^2^ = 0.72), discrimination (Harrell's C = 0.79) and calibration (calibration slope = 0.96) (24). Our calculator is based on simple sociodemographic variables (age, gender, ethnicity, age by gender interaction and ICD-10 index diagnosis) that can be easily accessed in clinical practice. It has been termed as “transdiagnostic” because it leverages several ICD-10 index diagnoses, and it can detect risk of psychosis across all diagnostic spectra (i.e., acute and transient psychotic disorders, substance abuse disorders, bipolar mood disorders, non-bipolar mood disorders, anxiety disorders, personality disorders, developmental disorders, childhood/adolescence onset disorders, physiological syndromes and mental retardation). This also represents one of the broadest transdiagnostic studies in psychiatry overall (38). The selection of predictors that were available in the local electronic health records was deliberately done with the view of facilitating its implementation in clinical routine at scale, which is an essential prerequisite to improve the detection of individuals at risk of psychosis. The predictors were preselected on the basis of a priori meta-analytical knowledge (39), a method which is recommended to develop robust prognostic models (31). In fact, the calculator is characterized by a significant clinical utility (net benefits) within a 1–50% range of threshold probability (individuals risk of developing psychosis by 5 years) (24). Such a range of predicted risk for psychosis is clinically meaningful since it is unlikely that a calculator would be needed to guide clinical practice for individuals with higher or lower predicted risks. The transdiagnostic risk calculator has also been implemented online (www.psychosis-risk.net) (24). This would allow its use in NHS Trusts that do not routinely employ electronic health records. An example of the output that is provided by the transdiagnostic risk calculator is appended in Figure 2. The core characteristics of the transdiagnostic risk calculator are appended in Table 1.

**Table 1:** Core characteristics of the transdiagnostic risk calculator.

Robust	It includes predictors that have been selected through a priori clinical knowledge
Pragmatic	It is agnostic with respect to etiopathology of psychosis
Cheap	It leverages predictors that are routinely collected by clinicians
Automatized	It can accommodate electronic health records as well as the manual entry of predictors
e-Health	It has been implemented online
Scalable	It can be used to screen large electronic health records
Optimisable	It can be further refined by the inclusion of other predictors

### Design

#### *In-vitro* Phase

**Figure 3:**
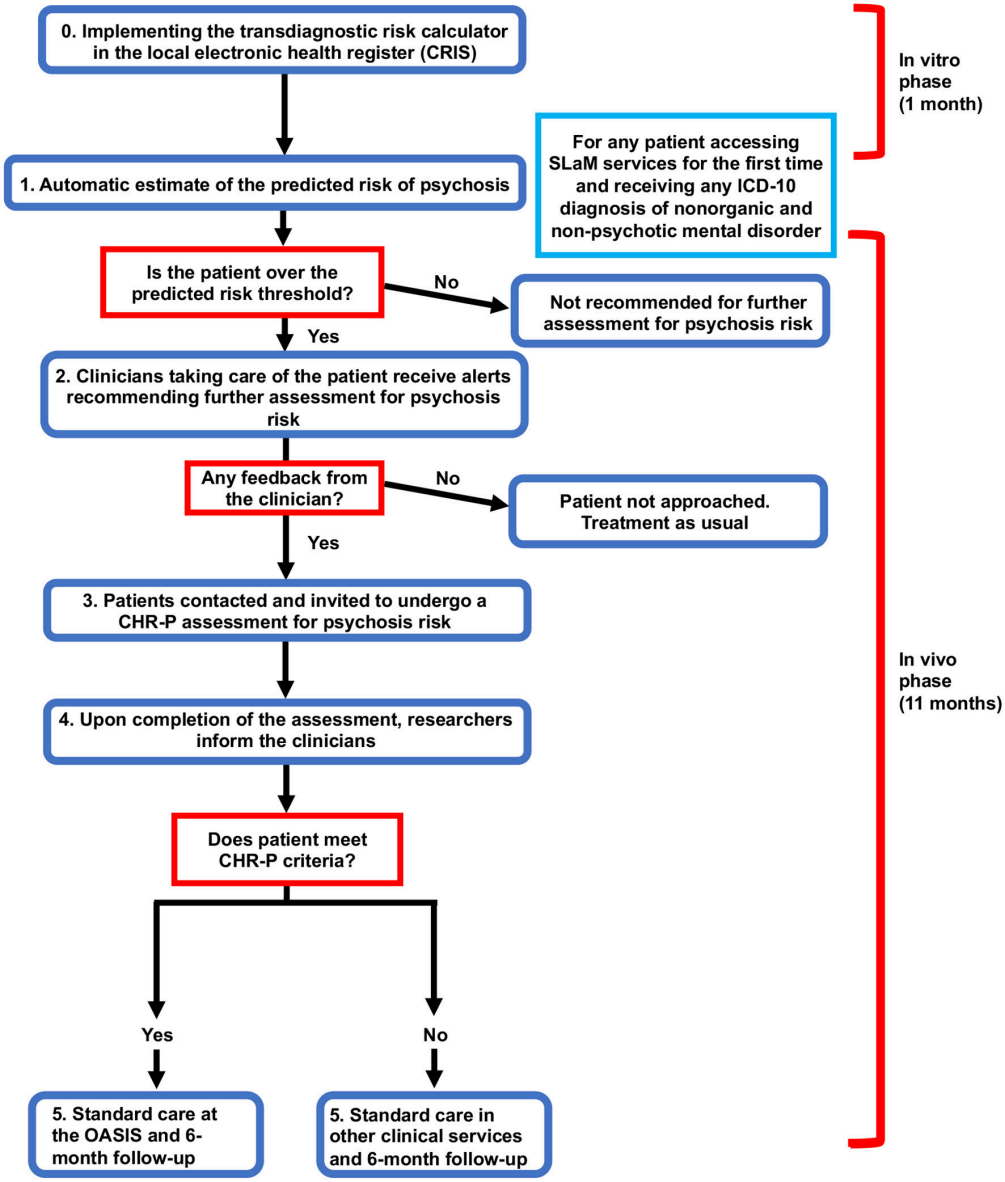
Flow chart of study design including the *in-vitro* and *in-vivo* phase.

The initial phase will have the transdiagnostic risk calculator integrated into the local electronic health register (step 0, Figure 3). This will involve several activities such as developing the prototype, addressing *in-vitro* feasibility problems associated with its implementation in SLaM clinical practice, and conducting clinician engagement work prior to initiating the *in-vivo* piloting. The team has already started initial work to prepare the implementation of the calculator. Firstly, we have approached the local NHS Trust IT facility (SL@M Connect) to discuss governance issues for using clinical material from the local NHS Trust. SL@M Connect has endorsed our study and will support it. Secondly, we have conducted data quality checks with the CRIS team to ensure that the resources needed are in place. Thirdly we have started the *in-vitro* phase by extracting preliminary data and running our calculator. We have also collaborated with the Center for Translational Informatics in order to fully implement the calculator in the local electronic health register. Anonymised data will be used during this phase to develop a prototype of the tool that can be automatised within the local electronic health register. Qualitative data will be collected to identify pragmatic barriers associated with the use of the transdiagnostic risk calculator. This will be collected through organizing two workshops, each composed of five SLaM clinicians. This phase would also tune the pilot threshold probability to be used in the next phase and address implementation challenges that have emerged from the *in-vitro* phase. This phase will be developed in collaboration with SLaM IT Connect and with the Center for Translational Informatics at the Institute of Psychiatry, Psychology, and Neuroscience. Approval for the *in-vitro* study was granted by the Oxfordshire Research Ethics Committee C. Because the data set is made up of de-identified data, informed consent is not required. Furthermore, during this phase we will try to complete a further external validation of the transdiagnostic risk calculator in an independent NHS Trust in the UK which is using CRIS. This is seen as an essential step to address the transportability of our transdiagnostic risk calculator across different clinical scenarios.

The *in-vitro* phase will last 1 month.

### *In-vivo* Phase

This phase will consist of a prospective feasibility study to test the real world usability of the transdiagnostic risk calculator to detect individuals at risk of psychosis at scale in clinical routine.

During this phase, a prototype will be made freely available to clinicians working in secondary mental health teams. In practice, clinicians will not be required to enter any new variables because all the predictors are already available as part of the standard clinical practice. In the first step (see step 1, Flow chart), the research team will screen potential patients meeting our study criteria using de-anonymised electronic health register data (CRIS). The data for these patients will be accessed by the study team to screen potential participants with the transdiagnostic risk calculator. The electronic health register will also be used to identify the responsible clinician for any individuals who are at risk as determined by the pre-defined threshold (established during the *in-vitro* phase).

The research team will then contact the responsible clinician through manual alerts (e.g., emails) and phone contact, recommending the patient be referred for a refined psychosis assessment. If an individual does not reach the threshold, the calculator will recommend no further assessment and standard care will be offered as usual. If possible, these alerts will be automatised during the study. During the second step (step 2, Flow Chart), the responsible clinicians will then decide whether to initiate the referral or not. A crucial outcome to be investigated will be the impact of different types of alerts on the clinicians' adherence on the use of the transdiagnostic risk calculator. If the individual is not referred for further assessment, standard care will be offered as usual. In the case the participant is referred for further assessment, the clinician will ask the patient if they consent to their details being given to the research team. If they do, the individual will be contacted by the research team and the procedure for collecting informed consent will be initiated.

If the patient agrees to participate in the study, they will then be invited to undergo a refined assessment (step 3, Flow Chart), which includes the standard CHR-P assessment. Specifically, we will use the combined Comprehensive Assessment for At Risk Mental States (CAARMS, version 12/2006) (3) and Structured Interview for Prodromal-Risk Syndromes, version 5.0 (SIPS) (40), clinician-rated and iPad version, that have been developed in our department as part of previous ongoing studies.

At the end of this assessment, the researchers would communicate the results to the responsible clinicians (step 4, Flow Chart). In case the individuals would meet the standard intake criteria for a state of risk for psychosis (i.e., a CHR-P state), they would be referred to the local Clinical High Risk service [the OASIS (9)] for standard care as recommended by the NICE (41). If the individuals do not meet the intake criteria for a CHR-P state, the referrer will be informed, and standard care provided accordingly. Overall, there will be no change in the current standard care for any participants. This prototype will be piloted in all local secondary mental health services present in the borough of Lambeth, Lewisham, Croydon and Southwark.

Participants will be reimbursed for their participation in the study at an hourly rate of £10. For patients under the age of 16, their time will be reimbursed with Amazon gift vouchers, again to the value of an hourly rate of £10 per hour. Table 2 lists the study procedures.

**Table 2:** Study procedures for individuals detected by the transdiagnostic risk calculator and referred by clinicians for an assessment for psychosis-risk.

		**Baseline**		**Follow-up**
	**Screening Visit**	**Day 1**	**Day 2 (2 weeks+- 3 days)**	**6 months +- 2 weeks**
Patient information and informed consent	**X**			
CHR-P assessment		**X**	**X**	**X**

*The in-vivo phase will last 11 months*.

### Follow-Up

Individuals who are selected through the transdiagnostic risk calculator, referred by their clinicians for a psychosis-risk assessment and who accept it will be invited again to a face-to-face clinical follow-up at 6 months. This will consist of the same measures acquired at baseline.

### Statistics

#### Sample Size

This is a feasibility study to investigate key implementation parameters for an electronic risk calculator. As such the study is neither designed nor powered to validate new tools or test specific hypotheses.

The primary outcome of the *in-vitro* phase is the development and integration of the transdiagnostic risk calculator in the local electronic health register. As such, no power calculation is made for the *in-vitro* primary outcome.

They key rate-limiting barrier toward a scalable use of the transdiagnostic risk calculator in the broader clinical scenario is the clinicians' adherence to the recommendations made by the calculator itself. Therefore, the primary outcome of the *in-vivo* phase of this study is the adherence of clinicians to the use of the calculator, defined by the proportion of clinicians who have responded to the prompts sent on the recommendation of the electronic risk calculator from SLaM secondary mental health care. The sample size calculation is therefore made for this primary outcome. In line with the NIHR guidance, our main outcomes are feasibility parameters, and sample size calculation is made for the expected level of precision (45). Assuming a predicted psychosis threshold of 5–10% (at 2 years), on the basis of the previous study (24), we expect to detect at least 120 at-risk individuals per 11 months recruitment in SLaM. Conservatively assuming that only half of SLaM clinicians would eventually respond to the alerts generated by the calculator, the anticipated sample size would allow us to have an acceptable (42) maximum margin of error of 0.1 (i.e., 95%confidence interval (CI) ±0.1) for adherence rates of clinicians >60%. The secondary outcomes of the *in-vivo* phase will measure other key feasibility parameters that are necessary to implement the calculator in the wider clinical routine: impact of different types of alerts on the clinicians' adherence to the recommendations made by the transdiagnostic risk calculator; raw number of referrals initiated from secondary mental health care clinicians for an assessment of psychosis-risk; qualitative reasons for any lack of clinicians' adherence.

#### Analysis

This is a feasibility study and it is not planned to test any statistical hypotheses with regard to any of the endpoints in a confirmatory sense. For the exploratory evaluation of our hypotheses, a two-sided 95% CI of adjusted treatment differences will be computed. However, the CIs will have to be interpreted in the perspective of the exploratory character of study, i.e., as an interval estimate for effects under these conditions. All statistical analyses will be performed using STATA version 14 (43).

### Participants

#### Inclusion Criteria

- Subject receiving any first ICD-10 diagnosis of non-psychotic mental disorder (including Acute and Transient Psychotic Disorders) at SLaM (borough of Lambeth) between April 1^st^ 2018 and March 28^th^ 2018;
- Aged ≥14;
- Subject with a good understanding of spoken and written English.

#### Exclusion Criteria

- Present or past diagnosis of any ICD-10 psychotic disorder [excluding Acute and Transient Psychotic Disorders (44)];
- Any evidence of organic condition that may be responsible for psychotic symptoms.

#### Withdrawal of Subjects

If an individual decides to take part in the study, they will still be free to withdraw at any time, without giving a reason. Their decision will not affect the standard of care they receive from any medical services at any time. Identifiable data already collected with consent would be retained and used in the study. No further data would be collected but the individual would be approached at follow-up.

### Outcomes

#### *In-vitro* Phase

- Primary outcome: to develop and integrate an automated transdiagnostic risk calculator into the local electronic health register;
- Secondary outcome: to externally validate the transdiagnostic risk calculator in an independent NHS Trust in the UK;

#### *In-vivo* Phase

- Primary outcome: adherence of clinicians to the use of the transdiagnostic risk calculator (proportion of clinicians who have responded to the prompts sent on the recommendation of the transdiagnostic risk calculator).
- Secondary outcome: impact of different types of alerts on the clinicians' adherence to the recommendations made by the transdiagnostic risk calculator.
- Secondary outcome: raw number of referrals initiated from secondary mental health care clinicians for an assessment of psychosis-risk.
- Secondary outcome: qualitative reasons for any lack of clinicians' adherence.

### Data Management

#### Type of Study

This is not a randomized clinical trial but instead a prospective cohort study. The *in-vitro* stage utilizes de-anonymised data from the local electronic case register, while the second uses a case-control design (prospective cohort study in SLaM).

#### Types of Data

The main experimental outcomes are quantitative and include the raw number of at risk cases detected by the calculator, the raw number of referrals made by clinicians, the raw number of individuals meeting CHR-P criteria, the raw number of individuals developing any ICD-10 psychotic disorder over time. Qualitative data will also be acquired from workshops conducted with SLaM clinicians during the *in-vitro* phase and from SLaM clinicians contacted during the *in-vivo* phase (in case of lack of adherence).

#### Format and Scale of Data

Data will be stored in standard formats using standard software for the field allowing easy sharing with other scientists as well as maintaining long-term validity.

#### Data Access

Data will only be accessed by the research team. Physical data will be stored in a locked drawer at OASIS with access restricted to the research team. Information collected from participants during the clinical investigation will be treated confidentially. The researchers will collect data and transfer it without recording the patient's name or date of birth but coded with a subject identification code. Therefore, data is not directly traceable to individual subjects. A subject identification code links the data to the individual subject. The code will be safeguarded by the responsible investigator at the site; the key to this code (subject identification code list) will be kept at the site, with limited access by study team members only.

#### Data Security

Privacy laws and regulations will be adhered to during all procedures related to this study. The collection and processing of participants' personal information will be limited to what is necessary to ensure the study's scientific practicability and to assess the research questions. Information collected from participants during this clinical investigation will be treated confidentially.

The researchers collecting the data for this study will work under the direct supervision of the consultant psychiatrist of the OASIS team (Paolo Fusar-Poli) and who will ensure there is no breach of confidentiality.

Once recruited to the research, the participants will be allocated a participant ID number which will be attached to all research documentation along with their initials and date of participation. Any documentation, which would allow the identification of personal data, will be collected under the participant ID and will only be accessible by the researchers. All information collected during the study will be stored in a secure location at OASIS within a locked drawer only accessible by the researcher and the OASIS team. All data collected from the baseline assessment will be anonymised using participant ID and stored on a secure, encrypted, password-protected server. iPads will be based on existing technologies developed at King's College London and are in use for other research projects at the Department of Psychosis Studies.

Research data will be stored for a minimum of 5 years following the completion of the study. We intend to make use of the King's College London (KCL) Research Data Management system where data can be stored long-term.

### Ethics and Regulatory Approval

The Chief Investigator of this study undertakes to abide by the ethical principles underlying the Declaration of Helsinki and good practice guidelines on the proper conduct of research.

If the research is approved the Chief Investigator undertakes to adhere to the study protocol, the terms of the full application as approved and any conditions set out by review bodies in giving approval. The Chief Investigator undertakes to notify review bodies of substantial amendments to the protocol or the terms of the approved applications and to seek a favorable opinion from the main Research Ethics Committee (REC) before implementing the amendment. The Chief Investigator undertakes to submit annual progress reports setting out the progress of the research, as required by review bodies.

The CI will ensure that REC Favorable Opinion, Health Research Authority (HRA) approval, and SLaM Confirmation of Capacity and Capability will be in place before recruiting from SLaM. Should it be necessary to add research sites at a later stage, the sponsor will be approached to review an amendment for submission to the HRA, and Confirmation of Capacity and Capability will be obtained from the new NHS sites before starting recruitment from research sites.

#### Consent Procedures in Minors

For potential participants who are under the age of 16 years old at the start of the study, informed consent should be provided by their legal representatives/parents, in line with the Declaration of Helsinki and International Conference on Harmonization-Good Clinical Practice (ICH-GCP). Their consent must represent the minor's presumed will and may be revoked at any time, without detriment to the minor. Whenever appropriate, the minor should participate in the informed consent process together with the parents. If the minor is deemed to be able to give assent to decisions about participation in research, the researcher will obtain this assent in addition to the consent of the legal representatives/parents. If the minor's assent is not obtained, it is recommended that this is documented with justification in the consent form which is signed by the legal representatives/parents and investigator. The minor's assent is not sufficient to allow participation in the study; informed consent of the legal representatives/parents is required. Consent from legal representatives/parents and assent from the minor should be sought at the same time. In any case, the minor will receive information according to its capacity of understanding regarding the study and its risks and benefits from staff with experience in minors. The explicit wish of a minor who is capable of forming an opinion and assessing this information to refuse participation will be followed; in such case, the minor can be withdrawn from the study at any time.

In case a minor reaches adulthood (age of 16) during the study, the researcher is obliged to obtain informed consent from this participant as soon as possible. Informed consent from legal representative/parents is no longer required, although it is recognized that an adolescent is still vulnerable and may require additional discussions and explanations.

In case the above-described procedures are not in line with any applicable local law or regulation, any deviations need to be discussed and agreed upon with the sponsor, as well as clearly documented.

If a minor or incapacitated subject does not want to participate, they will not be included in the study. This is also explicitly stated in the information letter.

#### Management of Disclosures and Distress

The content of the assessments can potentially lead to patients feeling distressed or disclosing sensitive information. There are guidelines in place for managing these incidents. If this occurs, the researcher will contact the patient's consultant or team manager to inform them. The responsible clinician will then offer the patient an assessment and treatment plan. For issues where the consequences are more imminent, the Accident & Emergency department at King's College Hospital will be contacted and appropriate treatment and support will be offered.

#### Quality Assurance, Data Handling, Publication Policy, and Finance

Names and contact details of participants will be kept on separate databases from experimental data, with anonymous subject codes referencing between the two. Data will be kept in accordance with study ethical approval, research governance and the Data Protection Regulation Act (2018). We will encourage access to the anonymised raw data by external collaborators within this framework, in accordance with the international policy on data preservation and sharing, while maintaining strict confidentiality for study participants. Encrypted data will be saved for long-term storage and sharing within the KCL infrastructure.

The Institute of Psychiatrym Psychology and Neuroscience (IoPPN) has a dedicated communications office which disseminates research findings via the media (press releases, expert comment proactive placing) and communications vehicles such as the King's website, and those of partner organisations such as SLaM NHS Foundation trust and other King's Health Partners.

The study has been externally reviewed and approved for funding by King's Health Partners.

## Discussion

We have presented an innovative implementation study protocol, applying a pragmatic, clinically-based, individualized, transdiagnostic, risk calculator to the NHS. To our best knowledge, this is the first implementation study of a risk calculator for clinical routine in the CHR-P field. Implementation studies are as scarce as essential (31). The proliferation of risk models in the CHR-P field as well as in psychiatry has occurred largely without appropriate attention to implementation challenges, resulting in many models that have little or no clinical impact (32). In fact, many more risk prediction models are published than are externally validated, and only a tiny minority of these is then implemented in the NHS (31). To achieve successful implementation, which is the true measure of a prediction model's utility, we considered that the end from the beginning of the model development process. Because our aim was to improve the detection of individuals at risk of psychosis, it was necessary to screen a large NHS Trust at scale. To achieve this goal, we selected predictors that were already collected by clinicians as part of their clinical routine. Furthermore, the requirement of simple variables for implementation increases the number of data sets that could be used for the external validation of existing models, a current gap in the implementation of risk prediction models in psychiatry. Because of these considerations, we believe that the study protocol here described can advance knowledge and foster translational precision psychiatry research. We hope that the pragmatic and operational nature of this protocol will guide future researchers, funders and ethics committees toward the development of implementation studies for psychiatric populations. We recommend this protocol as a starting point to guide future implementation studies for risk prediction models in populations at risk for psychosis.

We believe that the protocol described here can contribute to the development of solid risk prediction models that can be implemented in clinical routine.

## Study Status

The study status is ongoing, and recruitment for the *in-vivo* phase commenced on 1 August 2018. The study has been approved by National Research Ethics Service (NRES) East of England - Cambridgeshire and Hertfordshire Research Ethics Committee (Ref: 18/EE/0066).

## Author Contributions

PF-P designed the study and the grant applications in liaison with all of the other co-investigators. DO wrote the protocol, information sheets, ethical applications. DO and GS will acquire and analyse the data. PF-P drafted this manuscript. All authors read and approved the final manuscript.

### Conflict of Interest Statement

The authors declare that the research was conducted in the absence of any commercial or financial relationships that could be construed as a potential conflict of interest.
